# The Effect of Parent Training Programmes on Screen Time and Social Function in Children with Autism Spectrum Disorder

**DOI:** 10.21315/mjms2022.29.6.14

**Published:** 2022-12-22

**Authors:** Chai-Soon Khoo, Sathyabama Ramachandram

**Affiliations:** Child Development Clinic, Paediatric Department, Hospital Pulau Pinang, Malaysia

**Keywords:** autism spectrum disorder, screen media, media use, parent training, family media use plan, social function

## Abstract

**Background:**

Children with autism spectrum disorder (ASD) are susceptible to excessive electronic screen media (ESM) use. This study aimed to evaluate the effectiveness of a parent training programme in improving the screen time and social functioning of children with ASD.

**Methods:**

This pre-/post-test quasi-experimental study involved parents by providing them with structured education based on the American Academy of Pediatrics (AAP)’ screen time recommendations. In total, 259 children with ASD aged 3 years old–12 years old were eligible. Of those children, 26 were excluded due to comorbidities or taking medications. Additionally, 28 parents participated. Children’s screen time were recorded, and social behaviour was scored using the Social Responsiveness Scale pre- and post-intervention.

**Results:**

There were significant reductions in the average daily screen time of children with ASD after their parents attended the training programme (−51.25 min; 95% CI: −78.40, −24.10). In subgroups with reduced screen time, the treatment effect of the intervention was significant in improving the social responsiveness total score (−3.09; 95% CI: −5.96, −0.22), the social communication scale (−3.64; 95% CI: −5.91, −1.36) and the restricted interest and repetitive behaviour (RRB) scale (−5.27; 95% CI: −10.29, −0.25).

**Conclusion:**

Parental training is effective in reducing screen time and improving social functioning in children with ASD.

## Introduction

Electronic screen media (ESM) has unprecedented importance in the lives of children and adolescents with autism spectrum disorder (ASD), where it is primarily used for entertainment purposes over social communication (1).

In 2010, the American Academy of Pediatrics (AAP) (2) recommended that children over 2 years old of age use ESM for no more than 2 h per day. In 2016, the AAP recommended limiting parent-supervised screen use of high-quality screen programmes to 1 h per day for children aged 3 years old–5 years old (3). Although there are no screen time restrictions for those 5 years old–12 years old of age, parents are advised to place consistent limits on their children’s time spent using ESM and the types of ESM used while ensuring ESM do not replace adequate sleep, physical activity and other social norms essential to health (4). These screen time limits are applicable not only for typically developing children and adolescents but also for those with ASD who use mobile devices to communicate (5).

Children with ASD are at a higher risk of developing a propensity for ESM use than their typically developing siblings (6–11). ASD-related behaviours include increased attention to the screen, imitation of words and developing procedural knowledge about screen devices (7). Children with ASD who are less engaged in social and physical activities are inclined to opt for screen use over other leisure activities (8). Teenagers with ASD have been found to have higher rates of problematic ESM use, which may be due to impulsivity and deficits in behavioural inhibition (9). Parental preference for more screen time as a means of regulating their children can further aggravate high ESM use (10–11).

We hypothesised that children’s screen time could be reduced with parental understanding of the principles behind the suggested guidelines. Given that there are no published data on ESM use among Malaysian children regarding screen time education, we aimed to create awareness among parents of Malaysian children with ASD through parent education based on the AAP’s guidelines and family media use plan (FMUP) (2, 3). We also aimed to investigate whether a reduction in screen time through parental intervention indirectly improved the social responsiveness of children with ASD.

## Methods

### Research Design

This pre-/post-test quasi-experimental study involved parents of children with ASD who were diagnosed by a single developmental paediatrician using the Diagnostic and Statistical Manual of Mental Disorders, 5th edition (DSM-5) criteria (12). The Autism Diagnostic Observation Schedule, 2nd edition (13), a standardised observation tool, was administered to children who had concerning features but did not fulfil DSM-5 ASD diagnostic criteria upon history taking and clinical observation.

### Participants

The parents of 259 children with ASD aged 3 years old–12 years old were eligible to participate in this study through a Child Development Clinic, Hospital Pulau Pinang (CDC HPP) database search. Given the heterogeneity of the study population and the fact that ASD is commonly associated with other disabilities, the author established exclusion criteria to reduce confounding factors that could potentially bias the results.

#### Inclusion Criteria

- Parents of child with ASD
- A child with ASD aged 3 years old–12 years old
- The child attended the CDC HPP during the study period (1 January 2019–28 June 2019)

#### Exclusion Criteria

- Comorbidities, such as epilepsy or a visual/hearing impairment and/or
- On medications, such as antipsychotics, anticonvulsants and stimulants

### Parent Training

Detailed educational, developmental, medical and social histories were obtained through parental interviews during recruitment prior to the parent training. The intervention programme and its materials were prepared in three languages (English, Malay and Chinese) by the main author, who is proficient in all three languages. The first part of the parent training involved a lecture on the detrimental effects of excessive screen time and the AAP’s screen time recommendations pertaining to the child’s age group (Table 1). Principles involving screen-free zones in the naturalistic environment, device curfew, no screen time an hour before sleep and no ESM devices in the bedroom were taught to the parents. Additional training included how to choose interactive and educational media programmes and how to diversify ESM use in a way that promotes interaction, connection and creativity. The parents were advised to co-view, co-play, video chat and use learning apps appropriately, balance online and offline time, display good manners, become good digital citizens and develop awareness of online safety. Handouts were given at the end of the training.

The second part of the training involved individual sessions to organise the AAP’s FMUP (Table 1). The parents were first given a table according to the age of their child (3 years old–5 years or 6 years old–12 years old). The parents detailed their child’s typical day on the given table and were coached to complete a personalised media toolkit. Important health practices were incorporated into the table by adjusting the timetable according to the principles of the FMUP and attainment of 1 h of exercise and 8 h–12 h of sleep. The parents were guided individually to prioritise these health practices, consider other responsibilities such as homework, sports and time with friends, and determine how much spare time could be considered for ESM use.

### Measures

Measures of each child’s screen time and social behaviour were taken pre-intervention and 6 months post-intervention through parent reports.

The parents first reported average daily screen time on weekdays and weekends over the last month at home, daycare or school. The average daily time spent on various ESM (14) were recorded using a data collection form in minutes and in proportions of 5 days on weekdays and 2 days on weekends. The parents then completed Social Responsiveness Scale Second Edition (SRS-2) (15) pre- and post-intervention. The SRS-2 questionnaires were not translated into other languages to ensure the results’ validity. The main author served as the interpreter when required.

The SRS-2 is a standardised, 65-item scale that objectively measures the symptoms associated with ASD. The SRS-2 total score will indicate mild to severe deficits in reciprocal social behaviour that are clinically significant and impact on everyday social interactions. The scores from SRS-2 are categorised into treatment subscales regarding social awareness, social cognition, social communication, social motivation and restricted interest and repetitive behaviour (RRB). Additionally, the SRS-2 offers two DSM-5 compatible subscales: social communication and interaction and RRB. The SRS-2 can be used as a tool to monitor symptoms throughout the lifespan (15). A decrease in scale and subscale scores denotes an improvement in social function.

### Data and Statistical Analysis

Cross sectional sociodemographic information on the family was captured, and descriptions of ESM use were expressed in sums and percentages.

The data were analysed using the IBM Statistical Package for Social Sciences (SPSS) (16) version 26.0. Analysis of the paired *t*-test was used to assess children’s average daily screen time, SRS-2 total score and SRS-2 subscale scores pre- and post-intervention. Other outcomes of the SRS-2 total score and treatment subscale score differences were measured in subgroups of children who achieved the AAP’s goal of average daily screen time of no more than 120 min and a reduction in their average daily screen time. Post-hoc analysis was conducted in two additional subgroups: children who achieved the average performance of the intervention and children who achieved the average performance of the intervention with maintenance of their average daily screen time to no more than 120 min. A *P*-value of < 0.05 was considered statistically significant.

## Results

**Figure 1:**
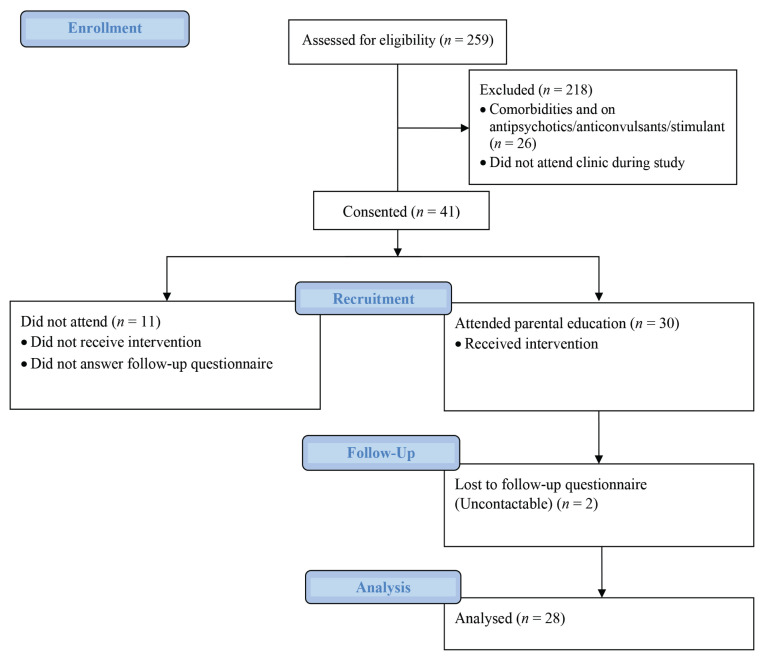
Flow diagram depicts the sequential progression of study from patient recruitment to data analysis

A total of 259 parents of children with ASD were eligible for this study. Among them, 41 parents attended the CDC HPP between 1 January 2019 and 28 June 2019 (Figure 1) and gave written consent, and 30 attended the parent education programme. Two families did not complete the post-intervention questionnaire due to non-attendance.

### Outcomes

The 30 parents who attended the parent training were from three main ethnic groups in Malaysia. The children’s male to female ratio of 4:1, with a mean age of 7.5 years old (Table 2). Regarding education, 15 parents obtained Bachelor’s or Master’s degrees, 10 had a Diploma and 5 had secondary school education. There were 12 families each in the low- and middle-income groups and 6 families in high-income group.

Regarding ESM use, the majority of the study population (two-thirds) spent no more than 120 min on ESM every day, whereas one-third had a daily screen time of more than 120 min (Table 3). There were four main categories of ESM: i) television; ii) computers; iii) mobile devices and iv) video game players. More than two-thirds of the children used at least two types of ESM on a daily basis. Mobile device and television were clearly the preferred choices, followed by computers. The majority of the children watched videos, followed by video game play using mobile devices. None played on consoles or handheld video game players.

Six months post-intervention, the ESM preferences remained somewhat similar, but more families adhered to the AAP guidelines after parent training. However, the total number of families that co-viewed screens and co-played video games remained constant. Two children who previously exceeded the AAP recommendation achieved a screen time of less than 120 min. The intervention effect was more apparent when the screen time usage trend was further analysed (Table 4). For more than half of the children, their average daily screen time expenditure decreased, about two-thirds remained unchanged and 7% increased. Out of 16 children who reduced their average daily screen time, the majority (*n* = 13) achieved an average performance of the study intervention (no less than 51 min) and 3 had a screen time reduction of less than 51 min.

A paired *t*-test was conducted to compare the total screen time and SRS-2 total score and subscale scores in 28 children with ASD pre- and post-intervention (Table 5). The average daily screen time before parent training (mean = 137.5 min, SD = 105.2 min) and after (mean = 86.3 min, SD = 68.3 min) revealed a significant reduction of 51.25 min with a *P*-value of 0.001. There was no significant reduction in the SRS-2 total score and subscale scores.

In the subgroup analysis based on the AAP daily screen time target and average performance of the intervention, there were no significant differences in SRS-2 total score and subscale scores in 22 children who kept their screen time to no more than 120 min (Table 6). However, for the 16 children who reduced their daily average screen time, an improvement in their social communication subscale score (−3.50; 95% CI: −6.91, −0.09) was identified. Likewise, 13 children who had a significant reduction in daily screen time of no less than 51 min showed a similar improvement in their social communication subscale score (−3.15; 95% CI: −5.44, −0.87). Further subgroup analysis of 12 children with significant average daily screen time reduction of no less than 51 min and maintaining it to no more than 120 min revealed improvement in their SRS-2 total score (−3.09; 95% CI: −5.96, −0.22), social communication subscale score (−3.64; 95% CI: −5.91, −1.36) and RRB subscale score (−5.27; 95% CI: −10.29, −0.25).

## Discussion

Elevated levels of screen use (2 h or more per day) have been shown to be significantly associated with behavioural and emotional difficulties (17–23). Our study showed that educating the parents of children with ASD on the AAP’s screen time guidelines and FMUP could lead to a significant reduction in children’s screen time and subsequent improvement in their social functioning by enhancing social communication and exhibiting less RRB. The results support recent evidence of a positive correlation of length of screen time to symptoms of ASD, specifically to an unusual interest in sensory input of taste, smell and touch, and negative correlation to language development (20).

The relationship between screen time reduction and improvement in social communication could be largely due to increased parental awareness of the negative effects of screen time on children’s level of physical activity (2) and replacement of screen time with developmentally appropriate, language-enriching learning opportunities (24). By reducing children’s screen time, opportunities are created to allow more social interactions with friends and family members and/or earlier intervention, which are crucial for developing language, communication and socioemotional skills (25–27).

Excessive ESM use and its causal link to the RRB symptoms of ASD can be bidirectional. Children with ASD may have an innate propensity for using media screen as a form of visual stimulation and elevated screen time can aggravate their dependence on ESM. This is supported by their tendency to develop procedural knowledge of screen devices (7). The debate on whether prolonged media screen use may alter sensory pathway development and perpetuate dependence on screens (8) requires further study.

The AAP’s FMUP is a helpful resource as well as a teaching tool through which paediatricians can provide information about the benefits and health risks of ESM. The positive impact of this intervention on screen time reduction and social function improvement can be attributed to the increased awareness of parents on these guidelines. In this study, only 13% of parents were aware of the AAP’s guidelines for ESM use for children at different ages and only 23% reported that they were given verbal screen time advice by doctors (28). The lack of awareness in the community presents an opportunity for paediatric health workers to impart knowledge to parents about the potential pros and cons of ESM use to reduce their children’s screen time and thus improve their social interaction (24). Paediatricians can explore and understand each family’s values and health goals and enhance their wellbeing through meaningful use of ESM (25–27). In comparison with other studies with more complicated methodologies, such as electronic monitoring systems or child education with parental involvement (28), this intervention is feasible and easily replicable, as the materials used are simple and the information is readily accessible on the AAP’s website.

This parental education programme’s approach is not only to reduce total screen time but also to encourage parents to promote balancing screen time with other important activities for health and wellbeing. Through provision of information on ESM and the facilitation of family-oriented screen time goals, we set parameters to promote healthy sleep patterns by encouraging healthy digital media accessibility and good family relationship.

The effectiveness of structured parent training on screen time reduction is proven and should thus be propagated through various platforms to achieve a population effect (i.e. a protocol or software application to reproduce similar results for the benefits of a larger population). An e-presentation or e-module as an effective tool during well-child clinic visits could be another option yet to be explored. During the ongoing COVID-19 pandemic, online parent training could also be a suitable virtual learning method.

## Study Limitation

This study had certain limitations. First, the author relied on parental reports, which are prone to recall errors and reporter bias (7, 29). Research is needed to develop objective measures for ESM use and to establish the reliability and validity of these measures in children with ASD.

In addition, this study is insufficient to power the study of potential confounding factors, including low socioeconomic status, single-parent households and a minority background, which have been associated with disproportionately high rates of screen time in other countries (30, 31). There may have been other influences on screen time practices over time which could be explored via a better study design, such as a case-control study.

Although the sample size in this study was small, it was comparable to international clinic-based studies with pre-/post-intervention design involving young children aged 6 years old–12 years old (32–35). Finally, another limiting factor was the short follow-up period of 6 months. A longer study period may be needed to monitor long-term efficacy of the parental training.

## Conclusion

Children with ASD are more susceptible to increased ESM use. This study demonstrated the effectiveness of parental training on screen time reduction and the improvement of children’s social communication and social behaviour. Parents are better supported in group educational therapy sessions, which increases the chances that parents will be persistent in reducing screen time. Future follow-up is necessary to assess the long-term effects of parental training, while we continue to encourage parents to explore a variety of enriching activities for children and adolescents with ASD.

## Figures and Tables

**Table 1:** Content of parent training programme

Parental education	

Group session	Individual session
Detrimental effects of excessive screen time	Draw out child’s typical schedule on a timetable
AAP screen time recommendation for specific age	Prioritise sleep, homework, sports and time with friends
Screen free zone, screen free time	
No screen an hour before sleep, mealtime, travel	
No media devices in the bedroom	
Choose interactive and educational media	To determine how much time is ‘left over’
Diversify ESM use	that may be considered for ESM use
Co-viewing, co-playing, video chatting	Balance online and off-line time
Observance of online etiquette	Bring home a printed AAP’s FMUP
Online safety and how to become a good digital citizen	

Notes: AAP = the American Academy of Pediatrics; FMUP = family media use plan; ESM = electronic screen media

**Table 2:** Sociodemographic information of children and parents

Children’s profile	*N* (%)	Mean (SD)
Sex		
Male	24 (80)	
Female	6 (20)	
Age (years old)		7.5 (2.5)
Ethnic background		
Malay	9 (30)	
Chinese	20 (67)	
Indian	1 (3)	
Parent’s educational level		
Primary school	0	
Secondary school	5 (17)	
Diploma/Equivalent	10 (33)	
Bachelor degree/Masters	15 (50)	
Household income and social grade		
Bottom (< RM3,000)	12 (40)	
Middle (RM3,000–RM15,000)	12 (40)	
Top (> RM15,000)	6 (20)	

**Table 3:** Comparison of ESM use practices pre- and post-intervention

ESM use	Pre-intervention (*n* = 30)	Post-intervention (*n* = 28)
Average daily ESM use	***n*** ** (%)**	***n*** ** (%)**
Less than 120 min	20 (66.7)	22 (78.6)
More than 120 min	10 (33.3)	6 (21.4)
Types of ESM		
** Television**	19 (63)	19 (68)
DVDs	2 (7)	2 (7)
** Mobile device**	21 (70)	16 (57)
Watching video	16 (53)	10 (36)
Playing games	11 (37)	10 (36)
Taking/looking at pictures	12 (40)	10 (36)
Video chatting	6 (20)	6 (21)
** Computer**	9 (30)	6 (21)
Watching video	6 (20)	3 (11)
Playing games	3 (10)	4 (14)
** Video game players**	0 (0)	0 (0)
AAP guideline adherence		
No television in bedroom	25 (83)	26 (93)
No charging of device overnight in bedroom	16 (53)	21 (75)
No media multitasking	18 (60)	25 (89)
No screen an hour before bed	8 (27)	17 (61)
No screen during short car ride	20 (67)	27 (96)
No screen during meals out of home	14 (47)	22 (79)
No screen during meals at home	14 (47)	22 (79)
Parents co-view screen	27 (90)	26 (93)
Parents co-play games	5 (17)	5 (18)

Note: Bolded parts indicated four major types ESM

**Table 4:** Comparison of daily ESM use post-intervention

Daily media use trend	*n* (%)
Static	10 (35.7)
Increment	2 (7.1)
Reduction less than 51 min	3 (10.7)
Reduction no less than 51 min	13 (46.4)

**Table 5:** Comparison of primary and secondary outcome measures of pre- and post-parent training

Outcome/Scale score	Pre- (*n* = 28)	Post- (*n* = 28)	Mean difference (95% CI)	*P*-value*

	Mean (SD)	Mean (SD)	Post- versus Pre-parent training	
Primary outcome				
Average daily screen time in minutes	137.5 (105.2)	86.3 (68.3)	−51.25 (−78.40, −24.10)	0.001
Secondary outcome				
SRS-2 total score	71.6 (8.6)	70.1 (7.8)	−1.43 (−3.60, 0.74)	0.188
SRS-2 DSM-5 compatible scales				
SCI	71.7 (8.8)	70.7 (7.8)	−0.96 (−3.02, 1.09)	0.345
RRB	68.0 (10.0)	65.7 (8.9)	−2.36 (−5.43, 0.72)	0.127
SRS-2 DSM-5 treatment subscales				
Social awareness	66.3 (10.0)	66.9 (9.1)	0.54 (−3.01, 4.08)	0.759
Social cognition	68.4 (9.2)	68.8 (7.6)	0.36 (−2.58, 3.29)	0.805
Social communication	72.1 (9.3)	70.1 (8.5)	−2.00 (−4.39, −0.46)	0.108
Social motivation	66.7 (11.1)	66.5 (7.7)	−0.14 (−3.74, 3.46)	0.936
RRB	68.0 (10.0)	65.7 (8.9)	−2.36 (−5.43, 0.72)	0.127

Notes: SD = standard deviation; SCI = social communication and interaction; RRB = restricted interest and repetitive behaviour;

* *P*-values were derived from paired *t*-test

**Table 6:** Comparison of mean social responsiveness score pre- and post-intervention

Scale/Subscale	Subgroup 1 (*n* = 22)			Subgroup 2 (*n* = 16)			Subgroup 3 (*n* = 13)			Subgroup 4 (*n* = 12)		

(T-score)	Mean difference	95% CI	*P*-value*	Mean difference	95% CI	*P*-value*	Mean difference	95% CI	*P*-value*	Mean difference	95% CI	*P*-value*
SRS-2 total score	−2.14	−4.70, 0.428	0.098	−2.75	−5.56, 0.60	0.054	−2.46	−5.10, 0.18	0.065	**−3.09**	**−5.96**, **−0.22**	**0.038**
SRS-2 DSM-5 compatible												
SCI	−1.59	−3.94, 0.75	0.173	−2.31	−4.92, 0.30	0.078	−1.77	−4.29, 0.75	0.152	−2.527	−4.95, 0.40	0.087
RRB	−3.27	−6.88, 0.34	0.073	−3.63	−8.30, 1.05	0.119	−4.23	−8.65, 0.18	0.059	**−5.27**	**−10.29**, **−0.25**	**0.041**
SRS-2 DSM-5 treatment												
Social awareness	−1.23	−5.00, 2.53	0.505	−1.31	−5.70, 3.07	0.533	0.54	−3.50, 4.57	0.776	−0.27	−4.31, 3.76	0.883
Social cognition	−0.27	−3.74, 3.20	0.872	−0.69	−3.15, 1.77	0.560	−0.62	−3.70, 2.47	0.672	−0.73	−4.02, 2.57	0.633
Social communication	−2.05	−4.82, 0.73	0.140	**−3.50**	**−6.91**, **−0.09**	**0.045**	**−3.15**	**−5.44**, **−0.87**	**0.011**	**−3.64**	**−5.91**, **−1.36**	**0.005**
Social motivation	−0.46	−4.04, 3.13	0.794	−0.50	−4.85, 3.85	0.810	0.39	−4.78, 5.55	0.874	−0.09	−1.36, −3.56	0.975
RRB	−3.27	−6.88, 0.34	0.073	−3.63	−8.30, 1.05	0.119	−4.23	−8.65, 0.18	0.059	**−5.27**	**−10.29**, **−0.25**	**0.041**

Notes: Subgroup 1: Children who achieved AAP goal of average daily screen time no more than 120 min; Subgroup 2: Children with reduction in average daily screen time; Subgroup 3: Children with significant reduction (no less than 51 min) in average daily screen time; Subgroup 4: Children who achieved significant reduction in and maintenance of average daily screen time to no more than 120 min; SCI = Social communication and interaction; RRB = Restricted interests and repetitive behaviour;

* *P*-values were derived from paired *t*-test; bolded parts indicated significant findings
